# Formation of dimers of light noble atoms under encapsulation within fullerene’s voids

**DOI:** 10.1186/s11671-015-0871-x

**Published:** 2015-04-17

**Authors:** Tymofii Yu Nikolaienko, Eugene S Kryachko

**Affiliations:** Faculty of Physics, Taras Shevchenko National University of Kyiv, 64/13, Volodymyrska Street, Kyiv, 01601 Ukraine; Bogolyubov Institute for Theoretical Physics, 14-b, Metrolohichna str., Kyiv, 03680 Ukraine

**Keywords:** Fullerene confinement, Noble atoms dimers, Bonding patterns, QTAIM

## Abstract

Van der Waals (vdW) He_2_ diatomic trapped inside buckminsterfullerene’s void and preserving its diatomic bonding is itself a controversial phenomenon due to the smallness of the void diameter comparing to the He-He equilibrium distance. We propound a computational approach, including smaller fullerenes, C_20_ and C_28_, to demonstrate that encapsulation of He_2_ inside the studied fullerenes exhibits an interesting quantum behavior resulting in a binding at shorter, non-vdW internuclear distances, and we develop a computational model to interpret these He-He bonding patterns in terms of Bader’s atom-in-molecule theory. We also conjecture a computational existence of He_2_@C_60_ on a solid basis of its theoretical UV absorption spectrum and a comparison with that of C_60_.

## Review

### Background introduction

At the recent lecture of Prof. Ihor R. Yukhnovskii ‘Phase Transition of the First Order Below the Liquid-Gas Critical Point’, partly published elsewhere [1], one of the authors of the titled work, E.S.K., has been actually impressed by a great vitality of the ingenious idea that lay behind a very simple equation of state, which Johannes Diderik van der Waals derived in his PhD thesis in 1837 and which won him the 1910 Nobel Prize in Physics [2], and that spread over centuries, the idea of the attractive dispersion force referred, after him, to as the van der Waals force. This force is responsible for the correction to the pressure in this equation of state and governs myriads of interactions appearing between atoms and molecules in a variety of chemical and biochemical processes [3-7]. Nevertheless, this vitality of van der Waals (vdW) interactions lies actually in that they continue - even after 177 years - to wonder: three recent events will serve as good examples.

The spectroscopic detection of the weakly bound van der Waals diatomic LiHe has been reported [8] in 2013. Actually, this system was predicted 14 years earlier, as existing with a single bound rovibrational state in the X^2^Σ ground electronic state characterized by the average bond length of approximately 28 Å and the binding energy of 0.0039 cm^−1^ (approximately 0.56 mK) [9]. Another surprise came in 2000 when the diffraction experiments [10] of molecular beam consisting of small clusters of He finally resolved the longstanding paradox with the van der Waals ^4^He_2_ dimer. The paradox - not yet then thought as that - started in 1931 when Slater and Krkwood performed the first calculation of the He-He potential [11], later corrected by Hirschfelder, Curtiss, and Bird in their famous book [12], and thoroughly reviewed by Margenau and Kestner [13], Hobza and Zahradnik [5], Kaplan [3], and Barash [14] (and the references therein, on the works of L. D. Landau school in particular). The importance of this interaction is hardly to overestimate since helium is the second most abundant element after hydrogen and the second simplest atom in the universe (see, e.g., [15]). The interaction between two helium atoms arises electrostatically, when an electric multipole on one atom creates a surrounding electric field that induces an electric mutipole moment on the other. In contrast to other molecular interactions, the van der Waals one is not related to a charge transfer - according to the Mulliken rule, the charge transfer is completely absent in the He-He interaction (see e.g., [16], p. 877) due to the enormous ionization potential of He equal to 24.5 eV, its small (in fact, negative) electron affinity, and very small polarizability *α* = 0.21 Å^3^. The latter make the He-He interaction extremely weak: the mean He-He internuclear distance (the bond length, in a sense) reaches 52 ± 4 Å and its binding energy 1.1 mK [10] (≈0.0022 kcal/mol, compared to quantum chemical accuracy of approximately 1 kcal/mol [17]), thus providing, first, an unusual inertness of He: Toennies [15] mentioned that only ‘a few compounds containing helium have been predicted [1], but none have been found,’ and, second, the breakdown of the Born-Oppenheimer approximation. In this lies the idea of the aforementioned paradox.

Another surprise came out from an unexpected side, from fullerenes [18]: in 2009, Peng and Wang et al. [19] developed the explosion-based method and prepared the endohedral fullerene He_2_@C_60_, which existence was confirmed in their mass spectrum experiments. It is not, however, absolutely clear how C_60_ enables to accommodate He_2_ dimer since its void diameter 0.7 nm = 7 Å is smaller than the aforementioned mean He-He internuclear distance, and thus, rules out that He_2_ dimer is still bonded therein. These authors claimed that the He-He bonding in He_2_@C_60_ arises due to the following mechanism: the repulsive interaction between two helium atoms keeps them away from the center, thus approaching each to C_60_ surface and establishing a charge transfer between He and C_60_. Altogether, this slightly distorts the C_60_ architecture that was detected in the peak recycling high performance liquid chromatography (HPLC) retention time. Only fewer computational works that have been done in parallel to this experiment mostly fell to agree with the latter and to explain it.

Our aim is to recover the agreement between experiment and theory by conducting a series of computations which include van der Waals effects and to offer the computational model behind the mechanism of bonding in He_2_@C_60_. The layout of the present work is the following. The ‘Methodological strategy’ section opens the methodological content of this work. The next section ‘Results and discussion’ focuses on discussing theoretical He-He and Ne-Ne bonding patterns and offers, and in the ‘Notes: computational experiment’ section, we give a definite computational evidence for the very existence of He_2_@C_60_ in terms of its theoretical UV absorption spectrum which is experimentally measurable. The work completes with thorough discussions and future perspectives.

#### Methodological strategy

All systems studied in the present work are divided into three categories: fullerenes, He- and Ne@fullerenes, and He_2_- and Ne_2_@fullerenes where the intermediate one is chosen as the reference origin to examine the He-He and Ne-Ne bondings in the last category.

#### Fullerenes

Fullerenes belong to the class of materials with a high ratio of surface to volume. According to the mathematical definition [20], *a fullerene is the surface of a simple closed convex 3D-polyhedron with only 5- and 6-gonal faces* (*pentagons and hexagons*). We assert that this surface/volume high ratio definitely predetermines a hollow cage architecture or void within a fullerene whose propensity is to accommodate (incapsulation or embedding) therein guest atom(s) or molecule(s) [21]. The latter system with the fullerene doped by atom is nowadays dubbed, after Cioslowski [22] and Schwarz and Krätschmer [23], as an ‘endohedral fullerene’ that originates from Greek words *ενδον* (‘endon’ - within) and *εδρα* (‘hedra’ - face of geometrical figure) [21].

**Figure 1:**
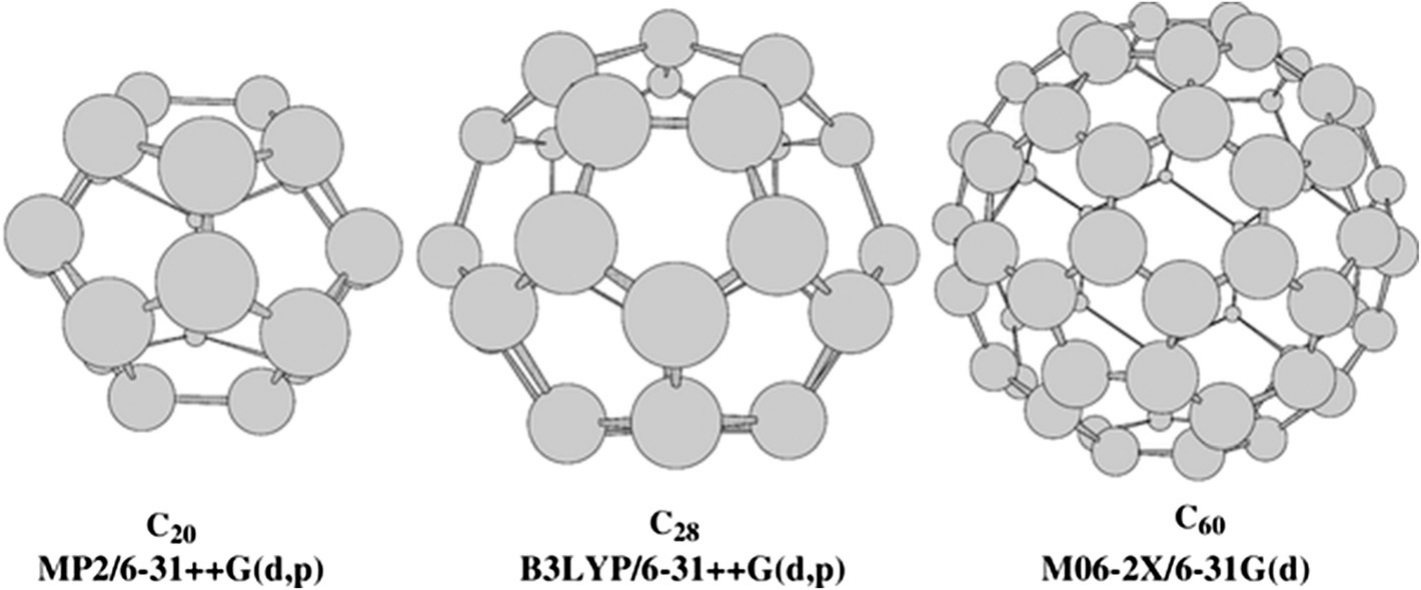
Studied fullerenes. Below their structures are indicated the corresponding computational levels invoked in the present work using Gaussian-09 package of programs [64] (keyword Int = UltraFine was invoked). C_20_ is the smallest fullerene consisting of 12 pentagons. Its HOMO-LUMO gap amounts to 0.96 eV [24]. C_28_ has a *Td* symmetry [25,26] and generates actually the smallest endohedral fullerenes M@C_28_ with M = Ti, Zr, and U [26,27]. For C_60_, we employ the M06-2X meta exchange-correlation density functional which takes into account a vdW-correction [65,66] and which was recently used in [67] for analogous tasks. As known, C_60_ consists of 12 pentagons and 20 hexagons. Each carbon forms a single double bond with the C-C bond length of 1.404 Å and two single bonds of 1.448 Å (note: MP2 ones are equal to 1.406 and 1.446 Å, respectively [68]).

The present work extends a class of He_2_@fullerenes to a variety of fullerenes that begins from the smallest C_20_ [24], intermediate C_28_ [25-27], and ends at the famous buckminsterfullerene C_60_ [18]. All of them are displayed in Figure 1 together with the invoked computational levels. The latter consist of two approaches based on the density functional (see e.g., [28] and references therein) and wave function (*ab initio*) theories. Notice that, according to [29], the MP2 and B3LYP predictions are very similar.

##### He@fullerenes and He_2_@fullerenes

Endofullerenes with encapsulated noble gas (Ng) atoms have been scarcely studied in the past, both experimentally and theoretically, compared to the first endohedral metallofullerene (EMF) La@C_82_, isolated in 1991 [30]. The reason is in that the Ng-encapsulation has very low yields and features a rather tedious separation from the host fullerene [21]. Though, the experiments on high-temperature decomposition of Ng@C_60_ ⇒ Ng + C_60_ have revealed the activation barrier of ca. 90 kcal∙mol^−1^ high [31,32] (see also [33]). On the theoretical side, it is worth mentioning the second-order Møller-Plesset perturbation and density functional computational approaches [29,33,34] to study Ng-endohedral complexes with C_60_-buckminsterfullerene [29,33,34] (see [21,35] for current reviews and references therein).

In the present work, Ng@fullerenes (Ng = He, Ne) and Ng_2_@fullerenes were studied at the same computational level as their cage fullerenes (see Figure 1). To reveal the bonding patterns in the He_2_@fullerenes, Bader’s ‘atoms-in-molecules’ (AIM) theory [36-38] was invoked since it provides a mathematically elegant approach [37] to describe a bonding. It should be noted beforehand that the theme on a chemical bond is rather fragile and subtle (see, e.g., Introduction in [39] and references therein). Once Bader [36] conjectured that one-electron density ρ(**r**), **r** ϵ ℝ^3^ of a given molecule should contain the essence of this molecule’s structure. Precisely, the topology of density is characterized by introducing the corresponding gradient vector field ∇_**r**_ρ(**r**) given by a bundle of trajectories as curves **r** = **r**(*s*) parametrized by some parameter *s* and satisfying the equation d**r**(*s*)/d*s* = ∇_**r**_ ρ(**r(***s***)**). The trajectories with zero gradient defines the zero-flux surface ∂Ω: = {**r** ϵ ℝ^3^| **n**(**r**) ∙∇_**r**_ ρ(**r**) = 0, where **n** = **r**/|**r**|}. This surface bounds a region in ℝ^3^ that defines a (topological) ‘atom’, or atomic basin. Two atoms are defined as *bonded* if they share a common interatomic surface. If follows from topology that each zero-flux surface contains a (3,–1)-type critical point **r**_c_ ϵ ℝ^3^ (CP) where ∇_**r**_ρ(**r**_c_) = 0 and where the Hessian matrix of *ρ* has two negative and one positive eigenvalues. The eigenvector of this Hessian matrix corresponding to its positive eigenvalue define two directions in which two trajectories of the ∇_**r**_ρ-field originate from the critical point forming a so-called ‘bond path’ connecting two attractors (which typically are atomic nuclei).

It is worth noticing that although AIM itself provides only a *definition* of a topological atom and does not provide a formal proof for its relevance to ‘chemical’ atoms, numerous examples demonstrate the fruitfulness of equating these concepts. In particular, bond paths have been shown to be a universal indicator of bonded interactions [40,41]. In the latter context, AIM is used in this work. Using Gaussian package, we obtained the electron density distributions at the corresponding computational level using the keyword ‘output = wfn’ and analyzed it with AIMAll package [42] to reveal all (3,–1)-type critical points and bond paths of the electron charge density distribution. A bond ellipticity that defines a measure of the extent to which a charge is preferentially accumulated in a given plane [30] was calculated as *ε* = λ_1_/λ_2_ − 1, where *λ*_1_ and *λ*_2_ are negative eigenvalues (|*λ*_1_| ≥ |*λ*_2_|) of the Hessian matrix ${H}_{ij}=\frac{\partial^2\rho }{\partial {x}_i\partial {x}_j}$ (*i*,*j* = 1,2,3) evaluated at the bond critical point. When possible transformations of a given molecular graph represented by a set of molecular bond paths are considered, it can be shown that ‘the ellipticity of the bond which is to be broken increases dramatically and becomes infinite at the geometry of the bifurcation point’ so that ‘a structure possessing a bond with an unusually large ellipticity is potentially unstable’ [37]. Therefore, the value of bond ellipticity can be considered as the measure of bond stability.

In conclusion, naturally anticipating the contribution of a vdW force into the bonding of the titled endofullerenes (see e.g., [43]), we also employed the ORCA package [44,45] using the density-dependent, non-local dispersion functional of Vydrov and Van Voorhis [46] in conjunction with the Ahlrichs’ TZV(2d,2p) polarization functions. Time-dependent DFT [47] was invoked within this package to calculate UV absorption spectra of C_60_ and He_2_@C_60_.

## Results and discussions

**Figure 2:**
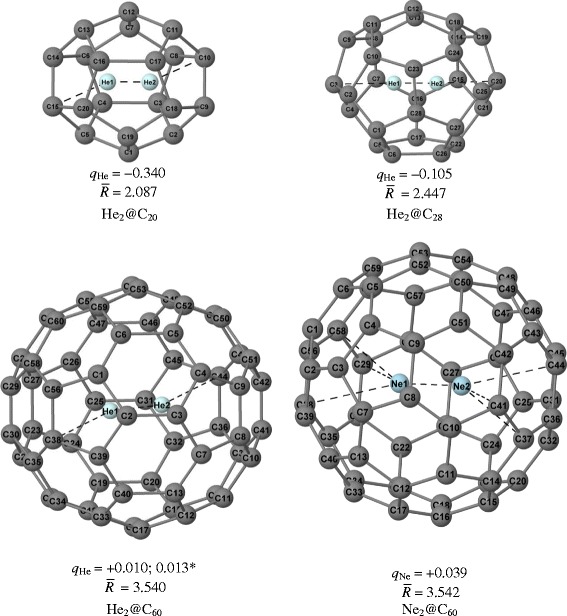
The calculated structure of endohedral fullerene Ng_2_@C_20_,C_28_,C_60_ (Ng = He, Ne), together with its atomic numbering.

The resulted structures of the studied He_2_@fullerenes together with some geometrical details are presented in Figure 2. We therefore envisage the following physics behind embeddings of the He_2_ diatomic into the voids of C_20_, C_28_, and C_60_ fullerenes. Let, for clarity and simplicity, limit ourselves by C_60_ and a single He atom.

Solid lines depict covalent bonds, whereas the dashed ones connect the atoms linked by a bond path according to the AIM analysis of the electron density distributions. *q*_Ng_ designates the Mulliken atomic charge of the Ng atom. The asterisk indicates the ORCA’s Mulliken gross atomic charge. Representative bond distances of the considered systems are presented in Table 1. $\overline{\mathrm{R}}$ (in Å) is the root-mean-square radii, $\overline{R}=\sqrt{\frac{1}{N}{\displaystyle \sum_{i=1}^N{R_i}^2}}$ (where *R*_*i*_ is the distance from the geometrical center to the *i*th carbon atom), of a given endofullerene with N carbon atoms: $\overline{\mathrm{R}}$ (C_20_) = 2.036 Å, $\overline{\mathrm{R}}$ (C_28_) = 2.427 Å, and $\overline{\mathrm{R}}$ (C_60_) = 2.540 Å.

**Table 1:** **Data of the AIM analysis of the electron density distribution in Ng**_**2**_ **@C**_**60**_ **(Ng = He, Ne)**

**Bond (A · · · B)**	***R***_**AB**_ **, Å**	***ρ*** ^**cp**^ **· 10** ^**2**^ **,** ***e*** **/** ***a***_**B**_^**3**^	**Bond ellipticity**
He_2_@C_60_			
He_1_ · · · He_2_	1.979^a,b^	1.26	3⋅10^−6^
C_38_ · · · He_1_	2.588	1.07	1.21
C_44_ · · · He_2_	2.588	1.07	1.21
He_2_^**+**^(^2^Σ_u_^+^):			
Present work	1.1881^c^		
MR-CI [69]	1.0816		
B3LYP [56]	1.1454^d,e^		
Expt. [57-59]	1.0806^f^		
Ne_2_@C_60_			
C38 · · · Ne1	2.649	1.57	8.31
C58 · · · Ne1	2.631	1.63	10.46
C29 · · · Ne1	2.638	1.60	5.53
C41 · · · Ne2	2.638	1.60	5.53
C37 · · · Ne2	2.631	1.63	10.46
Ne1 · · · Ne2	2.096	3.40	6⋅10^−5^
C44 · · · Ne2	2.649	1.57	8.31
C38 · · · Ne1	2.649	1.57	8.31

R_AB_ is the A · · · B-internuclear distance, *ρ* ^cp^ ≡ *ρ*(r_CP_), the charge density at the (3, –1) bond critical point (CP), and *a*_B_ is the Bohr radius. *ρ* ^cp^ qualitatively measures a strength of a non-covalent interaction. ^a^Other computational levels: BP86/TZVPP: 1.948 [39]; M05-2X/6-311G(d): 2.035 [70]. ^b^ *ν*(He-He) stretch in He_2_@C_60_ is centered at 531.0 cm^−1^. ^c^The calculated frequency in He_2_^+^ is equal to 1293 cm^−1^. ^d^ *ν*(He-He) stretch = 1359.9 cm^−1^. ^e^ *ν*(He-He) stretch = 1192.8 cm^−1^. ^f^ *ν*_expt_(He-He) stretch = 1698.5 cm^−1^ [57-59].

**Figure 3:**
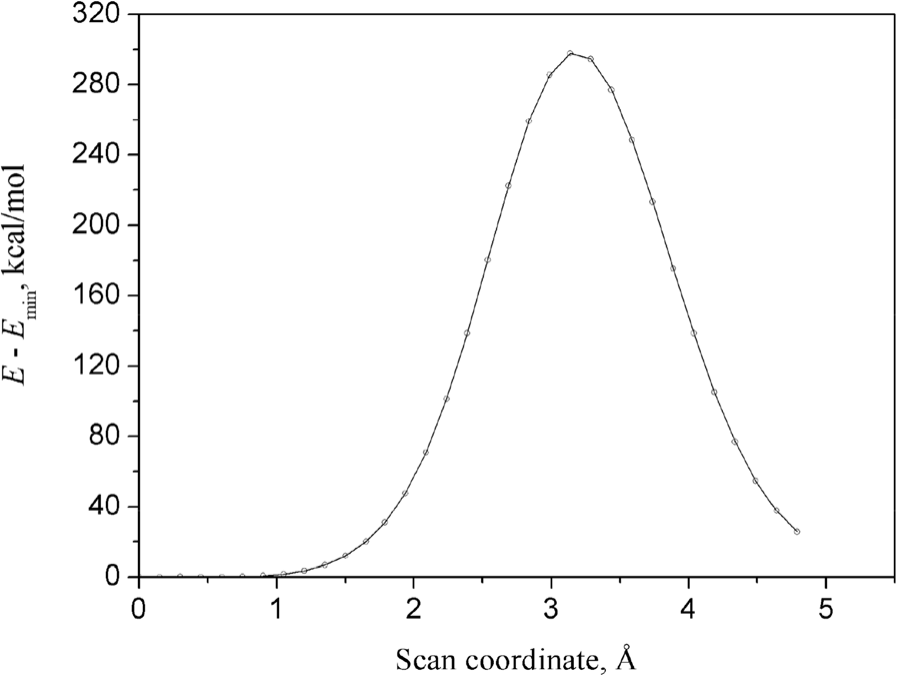
The barrier of embedding of He into C_60_ void obtained at the M06-2X/6-31G(d) computational level. Under the assumption of a frozen fullerene. The scan coordinate is the distance *R*(He_1_-C_38_).

The thought scenario of embedding of He into the C_60_ void from outside (a so-called exo-fullerene HeC_60_) includes a passage of He through a rather high barrier which profile is shown in Figure 3. This barrier of approximately 300 kcal/mol is rather high and ensures, on the one hand, a very large lifetime of He@C_60_. On the other hand, generalizing this barrier over the total fullerene surface, it obviously results in a confining void or confinement characterized by a high potential wall that ultimately results in a kinetic stability of He@C_60_ and its very large (practically infinite) lifetime, according to the transition-state theory (see e.g., [48] and references therein), though the recent calculations invoking PBE density functional with inclusion of the D3-type dispersion corrections [49] demonstrate a relatively weak binding of −2.434 kcal/mol.

**Figure 4:**
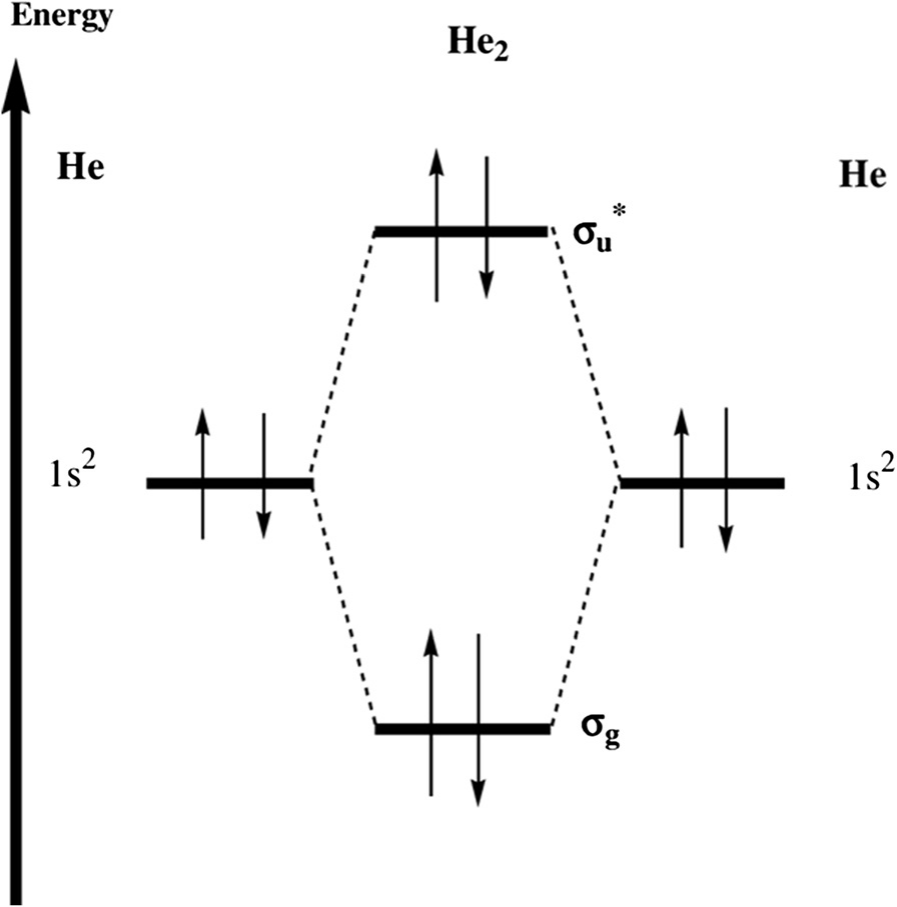
Formation of the bonding molecular orbital (MO) 1sσ_g_ and the antibonding MO 1sσ_u_^*^.

Lets imagine that at the next stage, the second He atom is added to the endohedral fullerene He@C60 with a single encapsulated atom of He. From Figure 2, it follows that the embedding of Ng_2_ (Ng = He, Ne) into buckminsterfullerene causes its slight swelling, indicated by $\overline{R}$, and a charge transfer from Ng_2_ to C_60_; however, the Mulliken charge *q*_Ng_, which measures this charge transfer, varies in fractions rather than in integers - note that variations of similar magnitude were observed in the work [50]. We suggest that a so-called ionic conjecture or the ionic model [21,35,51,52] is capable to explain this charge transfer by analogy with that taking place in EMF. In both cases, the host fullerene plays a role of an electron buffer [53]. Physics behind the charge transfer in He_2_@C_60_ is the following: approaching two ground-state atoms, say A and B, of He, whose two electrons occupy 1s_He_ atomic orbital, to each other results in formation of the bonding molecular orbital (MO) 1sσ_g_ and the antibonding MO 1sσ_u_^*^ (see Figure 4 and also [54]). The latter that is stronger, MO1sσ_g_, overlaps the LUMO (hole) of C_60_ that promotes a charge transfer He1sσ_u_^*^ ⇒ C_60_ –hole - the corresponding internuclear distances of 1.68 Å (Table 1) are smaller, a sum of the vdW radii which are equal to 1.4 Å for He and 1.7 Å for carbon, respectively [55]. This weakens the MO 1sσ_u_ and, in turn, strengthens the bonding interaction MO 1sσ_g_, converting He_2_ into fractionally ionized [He-He]^+0.02^ moiety. AIM properties of the latter sub-system are summarized in Table 1.

**Figure 5:**
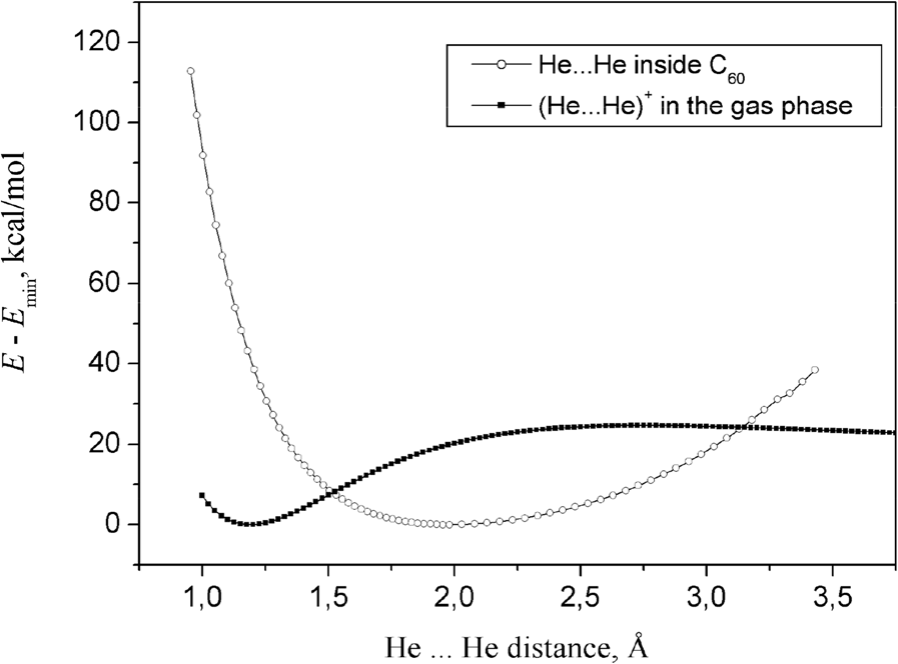
The He-He potential curves: [He-He]^+q^@C_60_ (open circle); the ground-state dihelium cation He_2_^+^ (filled circle; insert).

Interestingly, the He-He bond length in [He-He]^+0.02^@C_60_^−0.02^ contracts to 1.175 Å that is considerably smaller than the vdW-bond length in the He_2_ dimer. On the other hand, it rather well correlates with the equilibrium distance of the ground-state dihelium cation He_2_^+1^ equal to 1.2206 Å (cf. with the experimental value of 1.0806 Å [56-59]) obtained at the B3LYP/6-31G(d) computational level in the present work. We anticipate such behavior: the larger electron charge *q* (0 ≤ *q* ≤1) is removed from MO 1sσ_u_^*^, the stronger is bonding in [He-He]^+q^@C_60_^−q^. We continue this comparison of [He-He]^+q^ moiety embedded into C_60_ with the dihelium cation He_2_^+1^ in Figure 5 by comparing their He-He potential curves.

Finally, one can conclude from Table 1 and Figure 5 that in all studied complexes the He · · · He bond is much stronger than that between He and carbon atoms of the fullerene. In this regard, let us compare the He-He stretching mode ν_He-He_ in the studied endofullerene with ν_He-He_^expt^ = 1,698.5 cm^−1^ of the dihelium cation [57-59]: (a) in [He-He]^+0.02^@C_60_^−0.02^ ν_He-He_ contributes to the collective modes centered at 495.7, 504.8, and 531 cm^−1^. Note, for a purpose of comparison, that (b) in He_2_@C_20_ ν_He-He_ peaks at 2,380.6 cm^−1^, whereas in He_2_@C_28_ ν_He-He_ peaks at 1,682.1 cm^−1^; and (c) in [Ne-Ne]^+0.08^@C_60_^−0.08^ contributes to the collective modes centered at 440.6, 458.2, and 519.4 cm^−1^. A peculiar feature of He · · · C bond, especially in He_2_@C_20_ complex, is a rather large magnitude of ellipticity which, along with symmetry considerations, probably indicates that He_1_ · · · C_14_ and He_2_ · · · C_9_ bonds could also exist in He_2_@C_20_ complex (see Figure 2).

**Figure 6:**
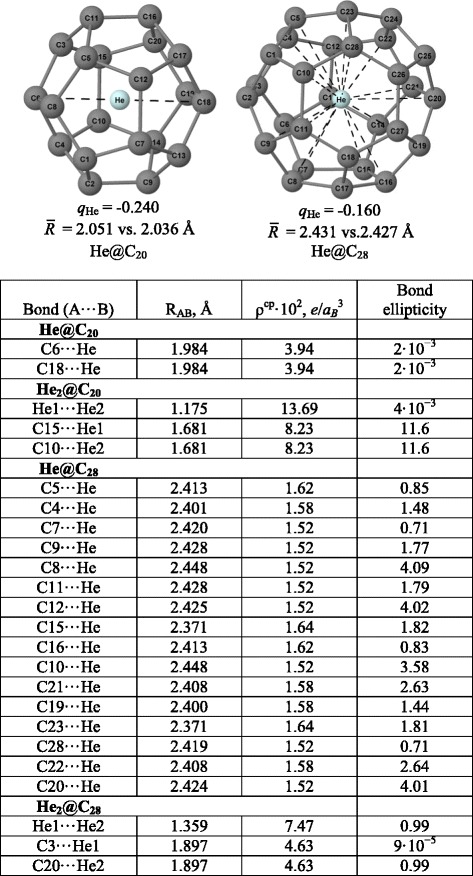
Characteristics of non-covalent interactions in Ng@C_20_ and Ng@C_28_ (Ng = He, He_2_) endofullerene complexes (for notations, see Figure 2 and Table 1).

Some representative properties of Ng_2_@C_20_ and Ng_2_@C_28_ are collected in Figure 6 where we add those for trapping a single noble-gas atom. Clearly, compared to Ng_2_@C_60_, the encapsulation of Ng_2_ into smaller C_20_ and C_28_ is governed by many other effects among which are worth noticing the steric effect and the following one:

- Overlap of the asymptotic tails of the electron densities of carbon atoms with that of He that may lead to negative Mulliken charges *q*_He_ since the corresponding internuclear distances of 1.98 and 2.37 Å are smaller than the sum of the vdW radii which are correspondingly equal to 1.4 for He and 1.7 Å for carbon [52]. To shed a light on this mechanism, we performed some additional calculations for the same complex geometries with the He atom replaced by the ‘ghost’ (defined as the ‘atom’ with the same set of basic functions and the zero nuclear charge). They show that (i) in He@C_20_, the ‘ghost’ He charge is −0.358 (vs. −0.240 for real atom) so that insertion of a single He into a void of C_20_ results in overlapping of electronic clouds and thus to negative Mulliken charge on helium; (ii) in He_2_@C_20_, the ‘ghost’ He atom acquires a Mulliken charge of −0.101 (cf. −0.340 for real atoms). It should be noted however that each of He_2_ atoms lies apart further from the center of C_20_ void as compared to the situation with the single He atom [28], so that the charge repelled by He_1_ can, in principle, induce an increase of population on He_2_ and vice versa.

### Notes: computational experiment

**Figure 7:**
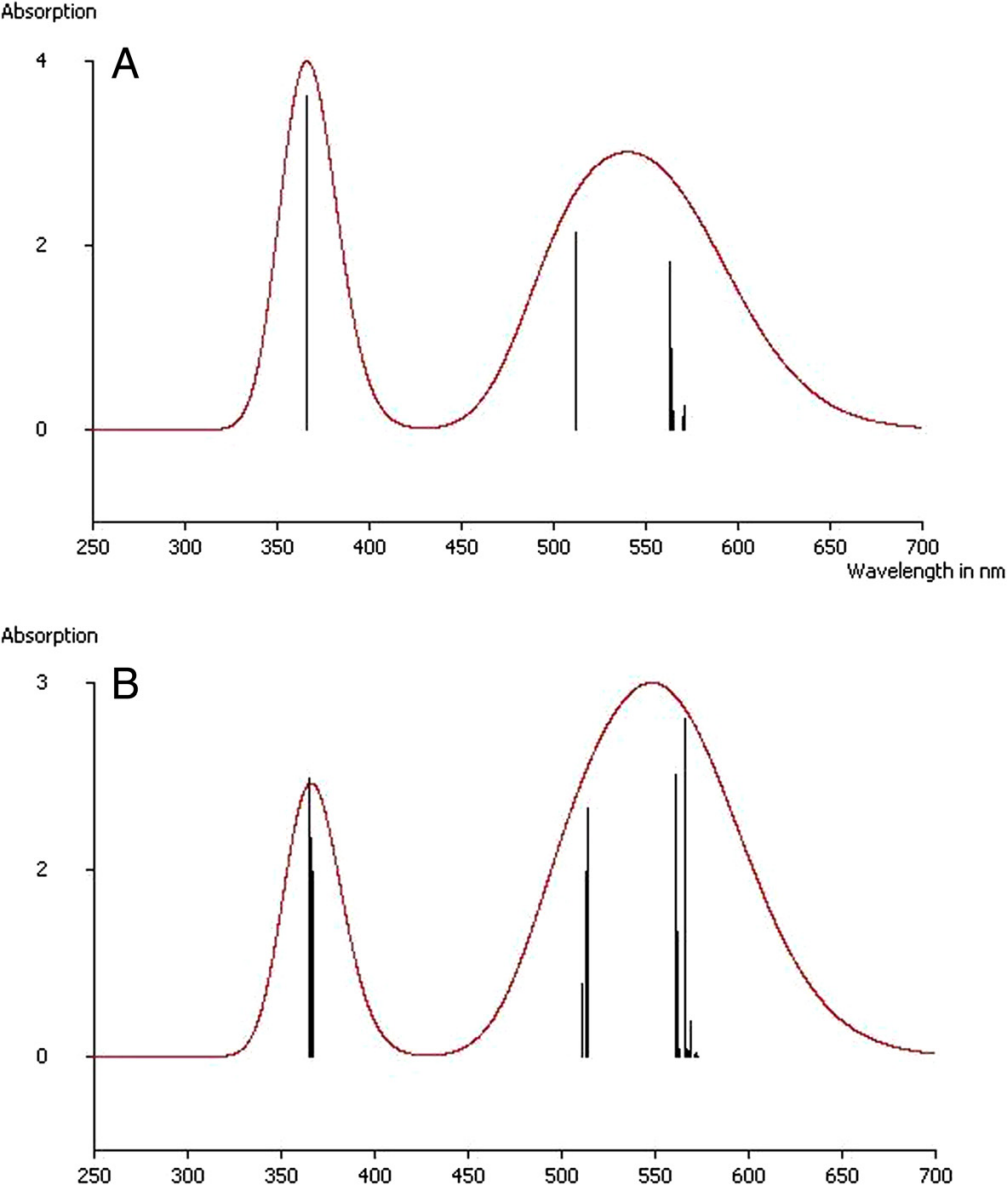
The UV absorption spectra: He_2_@C_60_ (top) and C_60_ (bottom) calculated within the TD DFT (width σ = 0.2 eV).

It is natural to view any computational model as a kind of experimental one, and hence, to extend all requirements, we usually impose on an experiment, on in its ‘computational’ cousin. Among them, one is reproducibility, the other is that an experiment should be treated as a test of a model or theory, and the third is the experiment’s capability to produce new data serving to test a given theoretical model. In the present work, the latter was chosen in the spectroscopic field: it is the UV absorption spectrum of He_2_@C_60_ system obtained in the present work using the TD DFT implemented in ORCA and presented in Figure 7 for its comparison with that of C_60_ by a straightforward analogy with the corresponding experimental spectrum, there are a strong peak around 300 nm and a very broad absorption between 450 and 600 nm [60]. Therefore, we may definitely conclude that if such the difference in the spectra of He_2_@C_60_ system and of C_60_ is experimentally observed, it is a solid argument in favor of the ionic mechanism of He_2_ bonding inside C_60_.

Glancing over Figure 7, our first impression is that the UV absorption spectra of He_2_@C_60_ system and of C_60_ are quite similar: two peaks, one is narrow, the other is quite broad - and such similarity we have already observed in the UV spectra of Kr@C_60_, also isolated by HPLC, and C_60_ [21,61]. On the other hand, this similarity emphasizes a distinguished difference of the studied UV spectra and therefore, the way to experimentally discriminate between the corresponding systems, He_2_@C_60_ and C_60_.

## Conclusions

After 30 years since the serendipitous discovery of fullerenes by Sir Kroto and co-workers [62], let us recall the statement by Ashcroft [63] that ‘The issue for C60 seems to go deeper.’ This is precisely what has been done in the present work which provides a solid computational basis for the existence of the buckminsterfullerene with the van-der-Waals-bonded He dimer which has been recently isolated in the HPLC experiments. A variety of its computational properties, from spectroscopic to the bonding ones, calculated by invoking Bader’s ‘atoms-in-molecules’ quantum theory, have been discussed and presented to identify its experimental ‘fingerprints’ and to reveal the mechanism of its bonding after trapping of He_2_ inside C_60_.
